# Visualization of coronary venous anatomy by cardiovascular magnetic resonance

**DOI:** 10.1186/1532-429X-11-26

**Published:** 2009-08-11

**Authors:** John F Younger, Sven Plein, Andrew Crean, Stephen G Ball, John P Greenwood

**Affiliations:** 1 Department of Cardiology, Royal Brisbane and Women's Hospital, Brisbane, Australia; 2 Academic Unit of Cardiovascular Medicine, University of Leeds, Leeds, UK; 3 Cardiac Magnetic Resonance Unit, Leeds General Infirmary, Leeds, UK; 4 Peter Munk Cardiac Center, Toronto General Hospital, Ontario, Canada

## Abstract

**Background:**

Coronary venous imaging with whole-heart cardiovascular magnetic resonance (CMR) angiography has recently been described using developmental pulse sequences and intravascular contrast agents. However, the practical utility of coronary venous imaging will be for patients with heart failure in whom cardiac resynchronisation therapy (CRT) is being considered. As such complementary information on ventricular function and myocardial viability will be required. The aim of this study was to determine if the coronary venous anatomy could be depicted as part of a comprehensive CMR protocol and using a standard extracellular contrast agent.

**Methods and Results:**

Thirty-one 3D whole heart CMR studies, performed after intravenous administration of 0.05 mmol/kg gadolinium DTPA, were reviewed. The cardiac venous system was visualized in all patients. The lateral vein of the left ventricle was present in 74%, the anterior interventricular vein in 65%, and the posterior interventricular vein in 74% of patients. The mean maximum distance of demonstrable cardiac vein on the 3D images was 81.5 mm and was dependent on the quality of the 3D data set. Five patients showed evidence of myocardial infarction on late gadolinium enhancement (LGE) images.

**Conclusion:**

Coronary venous anatomy can be reliably demonstrated using a comprehensive CMR protocol and a standard extracellular contrast agent. The combination of coronary venous imaging, assessment of ventricular function and LGE may be useful in the management of patients with LV dysfunction being considered for CRT.

## Background

Cardiac resynchronization therapy (CRT) is now an established treatment for chronic heart failure with broad QRS duration [1]. It requires the deployment of a left ventricular (LV) pacing lead to a branch of the cardiac venous system, usually via the coronary sinus. However this procedure is unsuccessful in 5–12% of patients for a variety of reasons including difficulty accessing the coronary sinus ostium, or lack of a suitable cardiac vein in which to position the LV lead [2]. Prior knowledge of coronary venous anatomy may prove useful to cardiologists both in the selection of patients suitable for CRT and for the guidance of LV lead implantation.

Demonstration of cardiac venous anatomy is recognized as an appropriate indication for cardiac CT scanning [3]. Coronary venous imaging with cardiovascular magnetic resonance (CMR) angiography has recently been described; however all previous studies have used either developmental pulse sequences or non-standard, intravascular contrast agents [4-6]. Late gadolinium enhancement (LGE) images, which can provide important complementary information for lead placement in CRT, were not acquired in these previous studies.

We sought to assess the feasibility and utility of using a three dimensional whole heart acquisition to visualize cardiac venous anatomy as part of a comprehensive CMR protocol that included myocardial function and viability scans and used a standard extracellular contrast agent.

## Methods

The CMR angiograms of 31 patients (18 male; mean age 58 ± 11 yrs) were retrospectively evaluated for their ability to demonstrate coronary venous anatomy. The scans had been performed for coronary artery imaging as part of a comprehensive research protocol in patients with known or suspected ischemic heart disease. Each scan also included assessment of LV function, myocardial perfusion (not reported here) and scar (late gadolinium enhancement). The investigation conformed to the principles outlined in the Declaration of Helsinki, was approved by the local ethics committee, and written informed consent was obtained from all patients. Inclusion criteria were, suspected coronary artery disease, sinus rhythm, and weight <110 kg (to ensure that study participants would be likely to fit within the magnet bore). Exclusion criteria were any contraindication to CMR imaging or adenosine infusion, resting heart rate >100 bpm, renal failure, previous coronary artery bypass grafting or recent acute coronary syndrome.

### CMR Protocol

All patients were examined supine in a 1.5 Tesla MRI scanner (Philips Medical Systems, Best, The Netherlands) equipped with master gradients (30 mT/m peak gradients and 150 mT/m/ms slew rate) and a 5 element dedicated cardiac phased array coil. Cardiac gating and triggering was performed via a vector ECG trace triggered on the R wave.

The position of the heart was determined by a rapid multislice, multistack survey scan in the transverse, coronal and sagittal planes. A low resolution coronary scout scan with a pencil beam respiratory navigator at the liver/lung border was performed to ensure adequate coverage of cardiac structures by the coronary imaging pulse sequence. High resolution coronary CMR angiography was then performed using a navigated 3D volume stack, with a steady state free precession sequence combined with fat suppression and T2 preparation prepulses (echo time 2.3 msec; repetition time 4.6 msec; flip angle 100°, SENSE factor of 1.7). The acquisition duration was individually adjusted to match the timing and length of the coronary rest period as determined from a horizontal long axis cine scan [7]. Using sufficient slices to cover the entire cardiac volume (whole heart coronary angiography – WHCA), 110–130 axial slices were acquired with a typical field of view of 360 cm^2 ^with an acquired spatial resolution of 1.18 × 1.18 × 1.80 mm^3 ^which was reconstructed to a resolution of 0.7 × 0.7 × 0.9 mm^3^. All WHCA scans were commenced within 5 minutes of intravenous administration of 0.05 mmol/kg of a gadolinium based contrast agent (Gadolinium-DTPA – Magnevist, Schering AG, Germany) for perfusion assessment. A further 0.15 mmol/kg contrast was administered prior to LGE imaging.

A short axis cine stack with 10–12 slices was acquired with a steady state free precession pulse sequence for quantitative LV functional assessment. LGE imaging was commenced 10–15 minutes after administration of the final contrast bolus (10–12 slices, inversion recovery segmented k-space gradient echo pulse sequence inversion time set to null signal from normal myocardium, spatial resolution 1.2 × 1.2 × 10 mm^3^).

### Analysis

The images were transferred to a workstation (ViewForum – Philip's Medical Systems, Best, The Netherlands) equipped with commercially available advanced segmentation tools (Coro3D) and volume rendering software and hardware. The coronary sinus (CS) was identified on the axial scans and the image quality was visually assessed using a three point scale (1 = high; 2 = adequate; 3 = poor) similar to that used previously for grading CMR coronary artery scans [8,9]. Subsequent results were grouped on the basis of the quality of the CS images; CS quality 1 where the axial images were of high quality, and CS quality 2/3 for sub optimal images. The data sets were then volume rendered and segmented to allow optimum visualization of the venous structures. The anatomy of the cardiac veins was studied on the 3-dimensional volume rendered image and in orthogonal planes using linked multi-planar reformatting and maximum intensity projections.

The dimensions of the CS and the presence of venous branches after volume rendering was recorded. The nomenclature used to describe the venous system is similar to that previously described [10], except that for simplification both posterior veins of the left ventricle and the left marginal vein were grouped together as left ventricular veins (LVV), as the area of venous drainage is similar. The diameter of the body of the CS on the axial and sagittal reformatted images, and the uninterrupted distance from the CS ostium to the most distal demonstrable end of a cardiac vein on the 3D image was measured. Presence of the anterior interventricular vein (AIV), posterior interventricular vein (PIV) and left ventricular branches were recorded, and the diameter of any left ventricular branches was assessed. The observer's confidence in the presence or absence of any LV branches was recorded on a 3 point scale (1 = reliable; 2 = probably reliable; 3 = not reliable).

Left ventricular mass and volumes were calculated by drawing the endocardial and epicardial contours on the end-diastolic and end-systolic images of the cine data sets and applying modified Simpson's rule. LGE images were analyzed visually for the presence of hyper-enhanced tissue in each of the LV short axis slices.

### Statistical analysis

The software program SPSS 14.0 (SPSS Inc., Chicago, Illinois) was used for statistical analysis. Continuous data are presented as mean ± standard deviation. Dichotomous variables are presented as absolute number and percentage. Analysis of variance was used to study differences between groups. The independent *t*-test was used to study differences between mean values and Levene's test was used to assess equality of variances.

## Results

### Baseline characteristics

**Figure 1:**
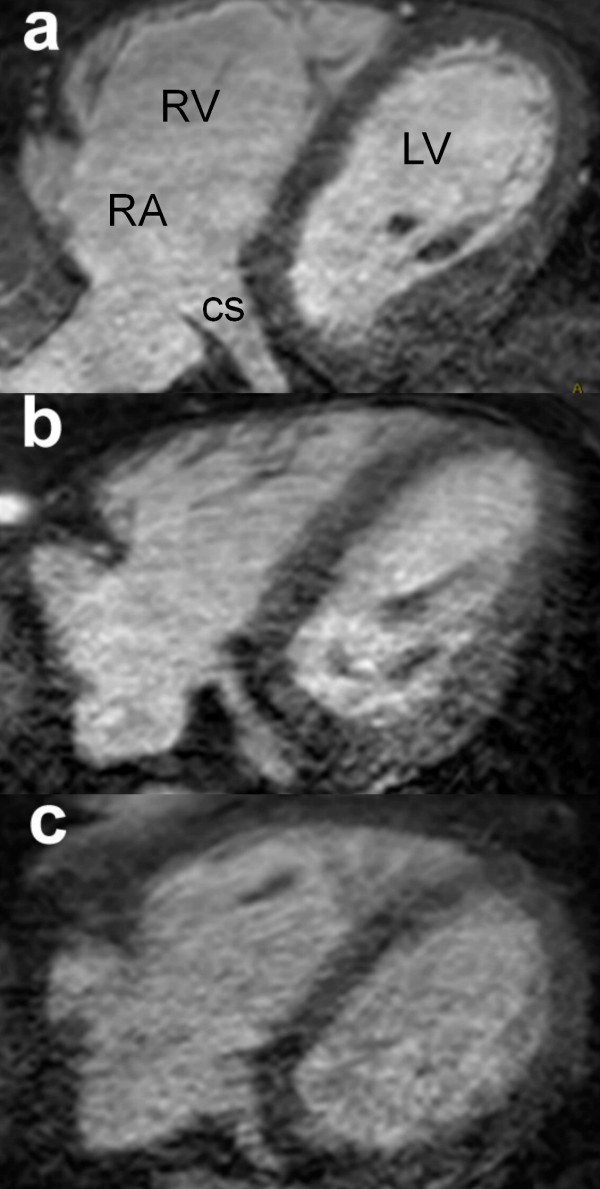
**Image quality**. The axial images from 3 patients demonstrating; a – high quality (CS1); b – adequate quality (CS2); c – low quality images (CS3). CS – Coronary sinus, LV – Left ventricle, RA – Right atrium, RV – Right Ventricle.

Table 1 summarizes the baseline characteristics of patients included in the study, all of whom had normal QRS duration on the electrocardiogram. Figure 1 shows axial images from 3 patients demonstrating; a – high quality (CS quality 1); b – adequate quality (CS quality 2); and c – low quality images (CS quality 3). There were no significant difference between patients with CS quality 1 images and those with CS quality 2/3 images, for any demographic factor including heart rate, age, BMI or LV ejection fraction. The groups mean LV ejection fractions were normal, but with a range of 24% to 65%.

**Table 1:** Characteristics of the study population

	All patients	CS1	CS2/3
Number	31	18	13
Male Gender	18 (58%)	10 (56%)	8 (62%)
Age (years)	58 ± 11	56 ± 11	62 ± 11
Weight (kg)	82 ± 13	82 ± 15	83 ± 10
Height (m)	1.69 ± 0.09	1.69 ± 0.08	1.70 ± 0.10
BMI (kg/m^2^)	28.7 ± 3.6	28.6 ± 3.7	28.9 ± 3.7
Non smoker	13 (42%)	9 (50%)	4 (30%)
Resting Heart Rate (bpm)	64 ± 12	61 ± 13	68 ± 10
Late Gd Enhancement	5 (16%)	2 (11%)	3 (27%)
MR quantitative data			
Ejection Fraction (%)	53.1 ± 7.3	54.6 ± 3.5	51.5 ± 10.5
Cardiac Output (l/min)	5.2 ± 1.0	5.2 ± 1.3	5.2 ± 0.9
EDV (ml)	157.9 ± 39.1	158.1 ± 35.5	157.7 ± 45.0
LV Mass (g)	92.6 ± 21.9	86.7 ± 24.5	100.8 ± 15.2

EDV = End Diastolic Volume. BMI = Body Mass Index. Gd = Gadolinium.

### Anatomic observations

**Figure 2:**
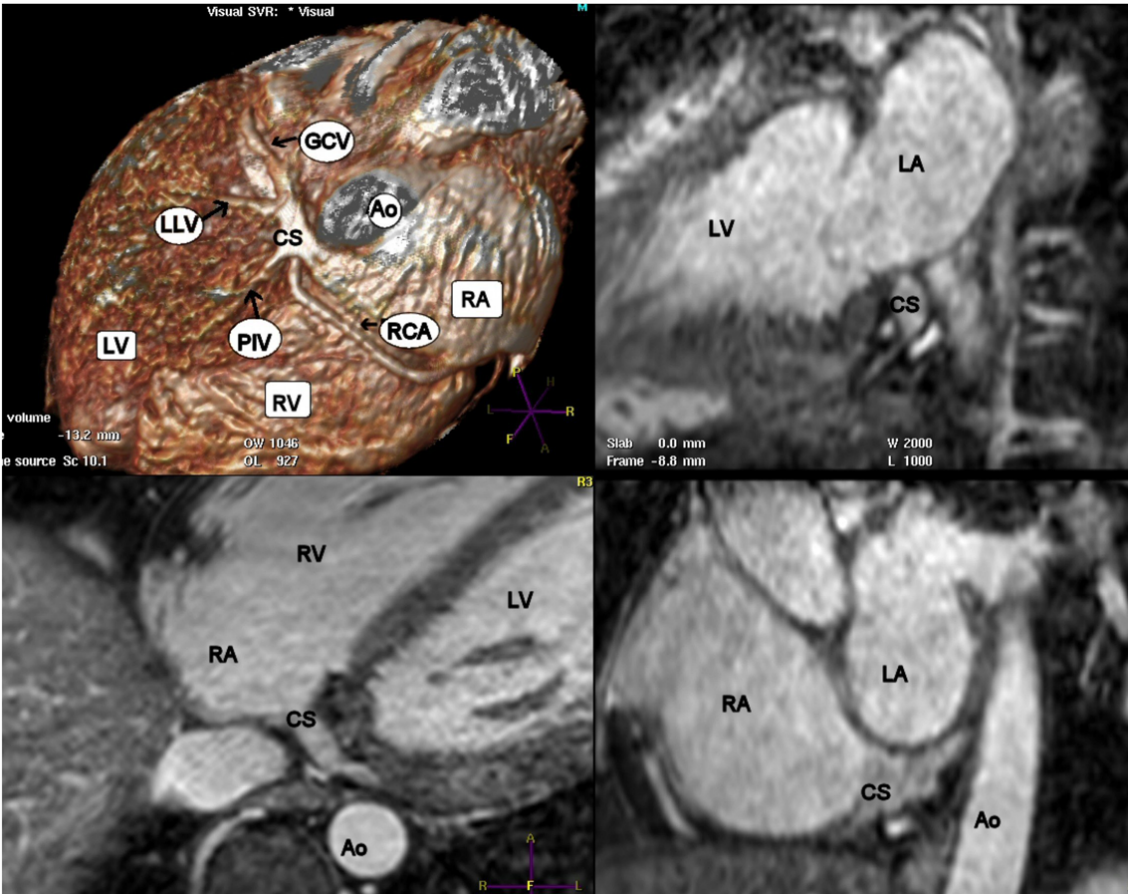
**Venous tributaries**. Volume rendered reconstruction and multiplanar reformatting in 3 orthogonal planes displaying the coronary sinus and venous tributaries. Ao = Aorta; CS = Coronary Sinus; GCV = Great cardiac Vein; LA = Left Atrium; LLV = Left Lateral Vein; LV = Left Ventricle; PIV = Posterior Interventricular Vein; RA = Right Atrium; RCA = Right Coronary Artery; RV = Right Ventricle.

Table 2 summarizes the data regarding coronary venous anatomy and quantitative measurements. The coronary sinus could be clearly visualized in all patients. In the entire study group, the LLV could be seen in 74%, the AIV in 65%, and the PIV in 74% of patients. In the high quality images (CS quality 1), the LLV was visualized in 89%, the AIV in 78% and the PIV in 83% of patients, whilst in the lower quality images (CS quality 2/3), a LLV could be seen in only 54% of cases, the AIV in 46% and the PIV in 62% of cases (Table 2). The differences between visualization of venous tributaries between CS quality 1 and CS quality 2/3 were only significant for visualization of the LLV. An example of volume rendered reconstruction and multiplanar reformatting of the coronary sinus and venous tributaries is shown in Figure 2.

**Table 2:** Coronary venous anatomy

	All patients	CS1	CS2/3
LV Veins			
Present	23 (74%)	16 (89%)	7 (54%)*
Diameter	2.6 ± 0.8	2.6 ± 1.0	2.5 ± 0.4
Confidence	1.6	1.1	2.2*
AIV	20 (65%)	14 (78%)	6 (46%)
PIV	23 (74%)	15 (83%)	8 (62%)
Max distance (mm)	81.5 ± 43.6	96.5 ± 41.8	60.9 ± 38.7*
CS Diameters (mm)			
Anteroposterior	9.9 ± 2.9	10.7 ± 3.1	8.8 ± 2.4
Superoinferior	11.5 ± 2.9	12.7 ± 2.9	9.8 ± 1.8*

*= p < 0.05 for a significant difference between CS1 and CS2/3. CS = Coronary Sinus. LV = Left Ventricle. AIV = Anterior Interventricular Vein. PIV = Posterior Interventricular Vein.

The confidence in assessing venous tributaries was significantly lower for the presence of LLV branches on the lower quality *vs*. the higher quality scans (mean score 2.2 *vs*. 1.1), although the diameter of any measured branches was similar in both groups (Table 2). In 2 patients no venous branches other than the CS and great cardiac vein could be seen (one of these was CS quality 1, the other was CS quality 3).

**Figure 3:**
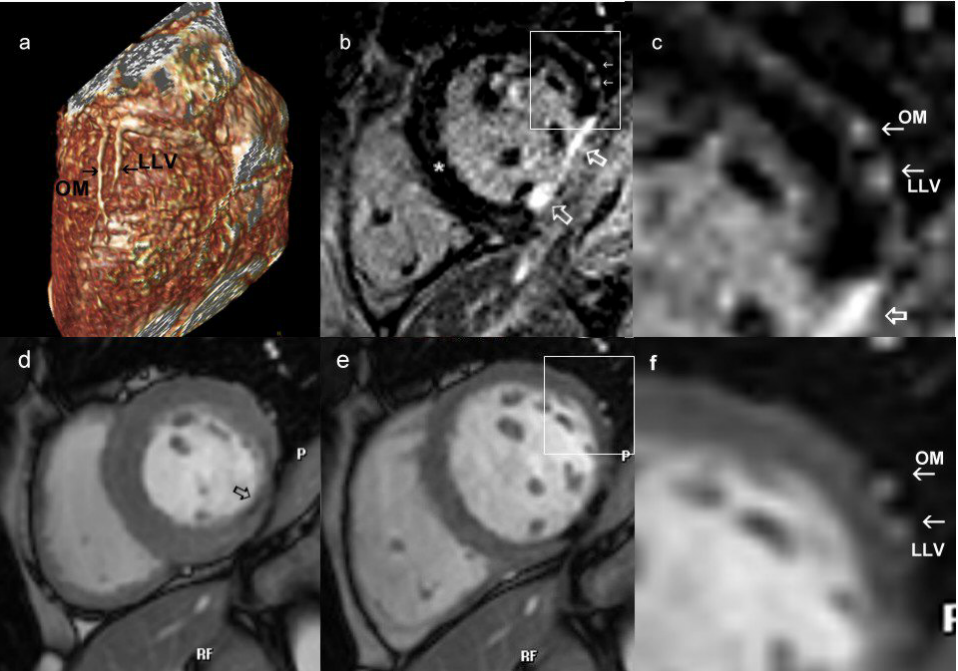
**Comprehensive CMR protocol**. 3a) The relationship of the OM branch of the circumflex artery and lateral vein can be seen on the volume rendered image. Knowledge of this anatomical relationship can be used in combination with late enhancement imaging, 3b) which shows area of scar (open white arrows) is present in the infero-lateral wall. A section of the lateral wall [panel] is enlarged in 3c) showing that the vessels do not over lie the scar. 3d) Systolic and 3e) diastolic frames from the cine images also reveal that the vessels, enlarged in 3f) are remote from the akinetic area (open black arrow).

In 5 patients there was evidence of hyper-enhancement on LGE images. Figure 3 shows how LGE and venous CMR images can be correlated to identify suitable positions for LV lead placement. In the image example, the lateral cardiac vein can be seen to lie just outside the area of lateral wall scar, and hence would be considered suitable for LV lead placement.

### Quantitative Measurements

The diameter of the coronary sinus was larger in the supero-inferior direction, than in the antero-posterior direction, in all groups as detailed in Table 2. Although there was no significant difference in the antero-posterior dimensions between the two groups, those in CS quality 1 had a significantly larger supero-inferior dimension than those in CS quality 2/3.

**Figure 4:**
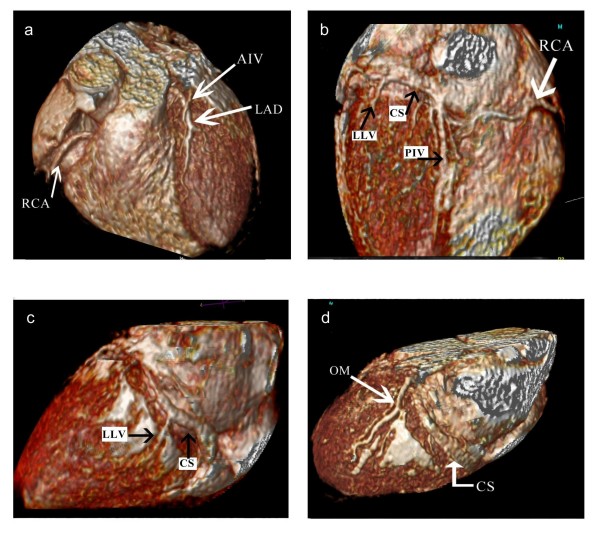
**Variations in venous anatomy**. Three dimensional volume rendered images from different CMR data-sets showing the anatomy of the cardiac veins. 4a – The AIV crossing over the LAD before a diagonal branch. 4b – The under-surface of the heart showing the PIV and a low LLV draining into the CS. 4c – A low LLV branch is seen. 4d – The CS is well demonstrated, but no tributaries are seen. High signal from pericardial fluid can be seen over the lateral wall. AIV = Anterior Interventricular Vein; PIV = Posterior Interventricular Vein; CS = Coronary Sinus; LAD = Left Anterior Descending Artery; LLV = Left Lateral Vein; OM = Obtuse Marginal Artery; RCA = Right Coronary Artery.

The mean maximum distance of demonstrable cardiac vein on the 3D image was 81.5 mm, 96.5 mm and 60.9 mm for all patients, CS quality 1 and CS quality 2/3 respectively. The difference between the 3 groups was significant (P < 0.05). There was large inter-individual variation in the lengths of visible vein, largely caused by overlying tissue obscuring the vein's path on the 3D images (Figure 4). In 4 cases, less than 10 mm of continuous vein could be demonstrated. However in 3 of these cases, distal tributaries of the venous system could be discerned on the axial images.

## Discussion

We have shown that CMR imaging of the coronary venous system can be performed as part of a comprehensive CMR protocol which includes myocardial perfusion, LV function and viability assessment and using a standard extravascular contrast agent. The techniques described in this study may be applicable to patients with heart failure undergoing CMR that are being considered for CRT.

CMR is already recognized as an important imaging modality for patients with heart failure, both in defining the aetiology and assessing the degree of LV dysfunction. In particular, patterns of scar tissue demonstrated by LGE imaging can be used to differentiate ischemic and non-ischemic origins, potentially avoiding invasive X-ray coronary angiography [11]. However CMR could also potentially provide information directly relevant to patients being considered for CRT. Firstly, a large scar burden, as detected by LGE imaging, is an important factor in predicting a lack of response to CRT, and has been proposed for inclusion in the selection process of CRT candidates [12]. Secondly, a recent study has indicated that CRT is less effective if the LV lead is placed in a vein overlying transmural scar in the postero-lateral LV wall [13]. Scar assessment with LGE is therefore an essential component of a CMR protocol in heart failure. Relating scar distribution to venous anatomy, as shown in Figure 3, potentially allows guidance of LV lead placement to areas of viable myocardium. The third application by which CMR could guide CRT is by providing prior knowledge of coronary venous anatomy. Location of the LV lead in a lateral vein, compared with lead placement in other locations, results in greater reverse LV remodelling and reduced diastolic dyssynchrony [14]. Hence patients with absence of lateral veins may not be ideal candidates for CRT.

The main appeal of using CMR imaging in patients considered for CRT is that in a single examination CMR could accurately assess left ventricular function, define venous anatomy, and assess both the aetiology of the heart failure and likelihood of response to CRT using total scar burden and location of scar tissue. Until recently, non invasive venous imaging was only possible using multi-detector CT (MDCT). However widespread use of MDCT is restricted by the necessity for large doses of both ionising radiation and iodinated contrast media. Furthermore, MDCT is generally restricted to assessment of the coronary vessels. The limited temporal resolution reduces the accuracy of MDCT for assessment of LV function when compared to modalities such as CMR or echocardiography [15], and, while MDCT has been proposed for viability assessment, this is not yet an established technique and leads to additional radiation exposure [16,17].

Several recent publications have demonstrated the ability of WHCA to delineate the course of the coronary veins in a three dimensional volume that can be reconstructed and volume rendered in a manner similar to MDCT [4-6]. The slow blood flow velocity and anatomic variability of coronary veins make them a challenging target for CMR imaging, so that intravascular contrast agents were used to enhance the coronary venous system in two of these three studies. However, intravascular contrast agents are not currently licensed for cardiac use and do not allow an assessment of LGE, which relies on contrast leakage into the extravascular space. Our data therefore complement the existing evidence for coronary venous CMR by showing that the coronary veins can also be imaged using a standard gadolinium-based contrast agent and in combination with myocardial function and LGE imaging. We expect that such combined assessment will be the most powerful clinical application for CMR in heart failure assessment and will distinguish it from MDCT. Importantly, measurements of the coronary venous system depicted in our study were similar to the results of previous studies using MDCT [10].

Our data also show that the delineation of the coronary venous system is dependent on the quality of the acquired data. For high resolution CMR whole heart imaging, data are acquired over many RR intervals (mean nominal scan time in our study was 5.32 minutes), so that the method is sensitive to changes in respiratory patterns and patient movement, as well as heart rate variation. The respiratory navigator can correct partially for bulk cardiac motion and arrhythmia rejection algorithms are available to limit the effects of heart rate changes, but in clinical practice around one third of WHCA studies are of impaired quality. These general limitations of CMR WHCA were also reflected in our study.

### Study Limitations

The CMR WHCA pulse sequence used in this study was designed to provide optimal visualization of the epicardial coronary arteries, and may therefore be suboptimal for demonstration of the coronary venous system. Further studies will be required to define the best methodology to reliably demonstrate cardiac venous anatomy by CMR. To our knowledge invasive venography, using retrograde contrast injection via the CS, has not yet been used to evaluate any non-invasive method of venous visualization. Without performing retrograde venography on all patients the true value of either CMR or MDCT to predict the anatomy of cardiac veins cannot be known. Hence the frequency with which CMR demonstrated venous branches in this study may be related both to the patient group or to the imaging modality and the relative contribution of each of these factors cannot be assessed. Finally the efficacy of CMR for coronary venous assessment specifically in patients with severe heart failure and broad QRS duration has not yet been assessed and may prove more challenging.

## Conclusion

Coronary venous anatomy can be demonstrated as part of a comprehensive CMR protocol that also includes late gadolinium enhanced imaging with a standard extracellular contrast agent. This may prove a useful addition to standard CMR in the assessment of patients with LV dysfunction who are being considered for CRT.

## Competing interests

The authors declare that they have no competing interests.

## Authors' contributions

All authors were responsible for study design, conduct and analysis, and all read and approved the final manuscript.
